# Silicone Breast Phantoms for use with Digital Imaging Elasto-Tomography Breast Cancer Screening

**DOI:** 10.1016/j.ohx.2025.e00707

**Published:** 2025-09-29

**Authors:** Francesca Gallaway, Henry W. Hall, Matthew Walker, Chloe Tattersfield, Stephanie Post, Jessica Fitzjohn, Cong Zhou, J.Geoffery Chase

**Affiliations:** Centre of Bioengineering, Department of Mechanical Engineering, University of Canterbury, Christchurch, New Zealand

**Keywords:** Breast cancer, Medical Imaging, Silicone, Material Properties

## Abstract

Breast cancer is the most common form of cancer and has the highest mortality rate among all types of cancer. The current best method to prevent deaths from breast cancer is by early detection. The gold standard for breast cancer detection screening is X-ray mammography. However, it has several limitations, including invasive radiation exposure and poor performance in younger women with dense breast tissue. Digital Imaging Elasto-Tomography is a novel breast cancer screening device using mechanical vibrations and computer vision algorithms to determine the actuated breast tissue surface motion and, from them, infer the elastic properties of underlying breast tissue. It leverages the 400-1000% stiffness contrast between health breast tissues and tumor tissues.

To further develop this device breast phantoms are required to test device software, hardware, use, and to quantify diagnostic algorithm performance on repeatable phantom test subjects. These phantoms need to have elastic properties appropriately representing realistic breast tissues and tumor tissues. The production of both asymmetric, realistically shaped and symmetric breast phantoms are detailed. Each of these phantoms is comprised of different silicone compositions representing different tissues, including a skin layer, healthy fat tissue, and cancerous tumour tissue. Several tests were performed to validate these phantoms, including compression testing to determine the elastic modulus and its location within reported human tissue stiffness value ranges.

## Graphical abstract

**Figure:**
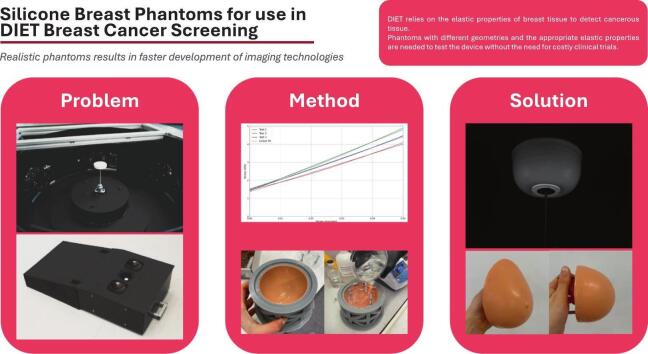

## 1 Hardware in context

**Specifications table**

**Table:** 

Hardware name	Silicone Breast Phantoms
Subject area	Engineering and materials science
Hardware type	Mechanical engineering and materials science
Closest commercial analog	*“*No commercial analog is available.”
Open source license	*CC BY 4.0*
Cost of hardware	*NZ$60 per phantom*
Source file repository	https://data.mendeley.com/datasets/hxm33bc9p5/3

Breast cancer is not only the most common cancer, but the main cause of death by cancer in women worldwide [1]. In New Zealand, breast cancer accounts for nearly 30% of new female cancers [2]. It is a priority area for health systems worldwide, and in New Zealand. Breast cancer survival rates are significantly improved by early detection [3]. The likelihood of tumours spreading to surrounding tissue or lymph nodes is reduced if they are identified early. Only 10% of tumours 10 mm or smaller will have spread to lymph nodes [4]. This likelihood of spread is notably less than the approximate 35% for 20 mm tumours, and 60% for tumours 40 mm or larger [4].

X-Ray Mammography is currently the best screening modality for large-scale population breast screening [1]. The discomfort associated with breast compression and the radiation exposure contribute to a low compliance rate with screening programs [1]. Tumours which are small or located deeper within the breast and/or near the chest wall are more difficult to identify [5]. Mammography also requires expensive infrastructure and skilled medical personnel to both facilitate screening and interpret diagnostic results [6]. The contrast in radio density between healthy and cancerous tissue is 5-10%, with a lower contrast in women with dense breast tissue [5]. It has been reported that mammography is less successful in younger women and those with dense breast tissue, which accounts for ∼50% of women [4]. In New Zealand, women under 45 are not recommended for mammography due to poor performance and the associated radiation risks. They are consequently diagnosed with later stage cancers and have worse outcomes, demonstrating breast screening inequity based on age [7,8].

Digital Imaging Elasto-Tomography (DIET) is an emerging breast cancer screening technology. It detects tumours by evaluating the contrast in elastic properties between healthy and cancerous tissue, which is between 400- 1000% [9]. This contrast is far larger than the 5-10% radio density contrast in mammography. This greater contrast enables higher potential resolution for detecting properties of breast tissue. DIET does not use radiation and is effective in women with dense breast tissue making it suitable for women of all ages. Thus, implementation of the DIET system has the potential to increase the accessibility and equity of breast cancer screening, improving health outcomes for all women.

DIET uses a configuration of cameras to reconstruct the breast surface motion when the breast is mechanically actuated with a steady state sinusoidal vibration. These motions are used to estimate the underlying tissue stiffness and damping properties [[10], [11], [12], [13]], where cancerous tissues would have high contrast in comparison to healthy tissues. However, development using human subjects alone to test and develop this technology, requires ethics approval, can be slow, and is more difficult due to inter-subject variability in response.

Thus, like ultrasound and MRI, the use of phantoms to test new devices, systems, configurations, or other features is important [[14], [15], [16], [17]]. Such phantoms are designed to mimic tissue properties relevant to the imaging modality excitation and approach. However, none exist for this low frequency, compared to ultrasound, vibration-based approach.

This work presents the methods for fabricating two different types of silicone breast phantoms, which would offer consistent, repeatable test beds if their mechanical properties are close to those of a real breast. The breast phantoms are designed for testing of the DIET machine. They could also be used in testing of other forms of breast or tissue mechanics research where accurate elastic properties are required. The work also presents a generalisable approach creating tissue phantoms across a physiologically realistic range to improve the generality of the DIET focused phantoms presented.

Two geometries of phantoms were developed including a symmetric, half sphere, shape and a more realistic human breast shape based on a prosthetic model [18]. The symmetric phantom was used to provide a benchmark test with a perfect convex shape. The realistic phantom provided concavities and asymmetries which can be used to test the surface reconstruction used in the DIET system. Comparison between the symmetric and asymmetric phantoms provides a better understanding of the performance of the DIET technology and identifies areas for improvement.

This article develops a method to produce symmetric and asymmetric breast phantoms which: 1) reflect the mechanical properties of real breast and tumour tissue; and 2) can be used to minimise the costs and time involved with human subject clinical trials for the purpose of developing and optimising DIET technology. Ultimately, the method outlined increases the speed of development by providing a realistic and repeatable test bed. As a result, these phantoms can contribute to bringing a more equitable breast cancer screening technology closer to market.

## 2 Hardware description

Breast phantoms are comprised of three different silicon compositions mimicking the three major areas of breast tissue types. Specifically, a skin layer, adipose tissue (body fat), and tumour tissue. The skin layer provides surface tension mimicking the movement of a real breast. An acrylic insert plate simulates the chest wall and provides a method of mounting the phantom to a plate.

A method for developing silicone breast phantoms had previously been examined but left several possible improvements to be made [[19], [20], [21]]. This research provided the basis for the development of the phantoms described in this article. The previous method produced phantoms considered too stiff to accurately model the material properties of real breast tissue. This method also used a core and cavity mould to create a skin layer. This approach made releasing the skin layer difficult without tearing or deforming the material.

The previous phantoms utilised A-341 Silicon Soft Gel purchased from Factor II Inc (Lakeside, AZ, USA). However, the company has since discontinued the product. Thus, the breast phantoms described in this article have a very different composition.

The method described in this work uses a simpler cavity mould that it is easier to replicate. The connectivity of the synthetic tumours to surrounding tissue mimicking materials was also improved during the development of this method. In particular, the tumours or inclusions in these phantoms are donut-shaped and cut from discs of stiffer material, which improves the connectivity to the adipose solution and better mimic the actual connectivity and load transmission between tumours and healthy tissues. Donut shaped tumours provide better connectivity by allowing adipose tissue to flow through the centre of the tumour holding it in place. This shape better mimics the interconnected nature of biological tumours. When the breast is actuated the tumour and adipose tissue are better coupled giving a more diffuse response than a perfect sphere. Their placement within the phantom can be of varying position and depth. Silicon pigment is added to the silicon mixture for the skin layer. Phantoms can thus be fabricated to represent women of all ethnicities, where visual light-based imaging, as used in DIET, can have variabilities across ethnicity.

The symmetric breast mould is a round, half-spherical shape with a 10.5 cm diameter and a volume of approximately 303 cm^3^. The average bra cup size of New Zealand women is B [22]. A 2010 study measuring the breast volume of women, and their corresponding bra size found that women sized with a B cup had a breast volume in the range of 250 cm^3^ - 400 cm^3^ [23]. Although it is acknowledged cup size is not homogenous in different band sizes, and there is significant variability in breast volume of different women, this data provides a reasonable range for the symmetric phantom design.

The asymmetric breast phantom model was developed from an existing model [18]. This model was chosen as it demonstrates the common concavity and asymmetry observed in breast shape, with a steeper slope on the medial (inside) of the breast where the DIET technology currently exhibits the most errors in motion measurement. It was altered to provide greater depth for the insert plate, include a seat for the top plate, and a draft angle of 3 degrees was added for removal after vacuum forming.

Since the DIET technology uses the stiffness contrast between cancerous and healthy tissue to detect tumours, it is critical this contrast is reflected in the breast phantoms. Real breast tissue is heterogenous and therefore challenging to replicate in detail. An accurate stiffness ratio between adipose tissue and tumours provides a sufficient imitation of real breast behaviour for testing purposes. Studies on the mechanical behaviour of breast tissue have shown the ratio of the elastic modulus of healthy to cancerous tissue ranges from 1:5 to 1:15 [[19], [20], [21]]. Consequently, a ratio of 1:5 was used as it is the smallest reported contrast and thus the most difficult to detect with DIET technology.

Compression testing of samples, as described in greater detail in Section 7, was used to determine the appropriate ratios of silicone materials. The silicone composition used a base soft gel silicone, Plastil Gel-0030, and a dilutant silicone oil, PMX-200. Different ratios of these two solutions were cured into standard sizes and compression tested. It was determined a ratio of 85% soft gel and 15% silicone oil was suitable for tumour tissue and 40% soft gel and 60% silicone oil was suitable for adipose tissue. This combination resulted in the tumours being 5 times stiffer than surrounding phantom tissue. Finally, 100% soft gel silicone was used for the skin layer.

The breast phantoms were designed for rapid development of DIET technology as a means of detecting breast cancer. They might also be useful in aiding research and design of other new breast cancer screening modalities. The overall approach presented can be generalised to create tissue phantoms for any need where the focus is capturing realistic tissue mechanical properties simply. There are currently no commercially available alternatives with the necessary mechanical behaviour for testing the DIET technology. Ultimately, the designed phantoms provide researchers with:

• A method simple to replicate with low-cost materials and resources.
• Breast phantoms that mimic the true elastic properties of healthy and cancerous breast tissue.
• An additional method of testing screening devices without the need for clinical trials.

## 3 Design files summary

All components required to produce the breast phantoms can be found in Table 1***.***

**Table 1:** Design files and their locations for the breast phantoms.

**Design file name**	**File type**	**Open source license**	**Location of the file**
*Symmetric Breast Phantom Mould*	*CAD (SLDPRT) and STL Files*	*CC BY 4.0*	*https://data.mendeley.com/preview/hxm33bc9p5?a=93ddd629-3074-4958-aae9-aba36da318d9*
*Symmetric Breast Phantom Insert Plate*	*CAD (SLDPRT) and DXF Files*	*CC BY 4.0*	*https://data.mendeley.com/preview/hxm33bc9p5?a=93ddd629-3074-4958-aae9-aba36da318d9*
*Symmetric Breast Phantom Top Plate*	*CAD (SLDPRT) and DXF Files*	*CC BY 4.0*	*https://data.mendeley.com/preview/hxm33bc9p5?a=93ddd629-3074-4958-aae9-aba36da318d9*
*Asymmetric Breast Phantom Model*	*CAD (SLDPRT) and STL Files*	*CC BY 4.0*	*https://data.mendeley.com/preview/hxm33bc9p5?a=93ddd629-3074-4958-aae9-aba36da318d9*
*Asymmetric Breast Phantom Insert Plate*	*CAD (SLDPRT) and DXF Files*	*CC BY 4.0*	*https://data.mendeley.com/preview/hxm33bc9p5?a=93ddd629-3074-4958-aae9-aba36da318d9*
*Asymmetric Breast Phantom Top Plate*	*CAD (SLDPRT) and DXF Files*	*CC BY 4.0*	*https://data.mendeley.com/preview/hxm33bc9p5?a=93ddd629-3074-4958-aae9-aba36da318d9*
*Base Plate*	*CAD (SLDPRT) and STL Files*	*CC BY 4.0*	*https://data.mendeley.com/preview/hxm33bc9p5?a=93ddd629-3074-4958-aae9-aba36da318d9*

The Mould, Model, Insert Plate and Top Plate design files are required for fabrication of the silicone breast phantoms. The Base Plate provides a means of storage for the phantoms and allows for easy positioning of them in the DIET machine.

## 4 Bill of materials summary

A bill of materials with the cost per item and the places these materials were sourced from can be seen in Table 2**.**

**Table 2:** The bill of materials summary for the breast phantoms.

**Component**	**Number/Qty**	**Cost per unit -currency**	**Total cost -currency**	**Source of materials**	**Material type**
*Clear Acrylic Sheet 4.5 mm*	*0.01m2*	*NZ$100/m2*	*NZ$1*	*https://frameless.co.nz/pages/acrylic-sheet-pricing*	*Polymer*
*Norski Mould Release Wax*	*5ml*	*NZ$134.56/L*	*NZ$0.7*	*https://www.norski.co.nz/products/norski-mould-release-wax?variant=39315506298973*	*Organic*
*Ease Release Mann 200*	*1*	*NZ$40/Can*	*NZ$1*	*https://www.resincraft.co.nz/mann-ease-release-200?srsltid=AfmBOorXwLwpFHLfD9M8PrNPBaWKUYCXyL9Ie8W_b-S11Oj_nvOLJtLb*	*Polymer*
*Platsil Gel-0030 Prosthetic Grade Silicone*	*289g*	*NZ$135.7/kg*	*NZ$39.2*	*https://www.resinart.nz/products/platsil-gel-0030-prosthetic-grade-silicone-1kg?srsltid=AfmBOoojuGJN91dj5ZTV8cQJ0prZoZ6yAxh4AaPZc-jDulKxM7CLli2-*	*Polymer*
*PMX-200 Silicone Fluid*	*241g*	*NZ$62.8/kg*	*NZ$15.1*	*https://trulux.com/products/xiameter-pmx-200-silicone-fluid-50cs/?_pos=2&_sid=020d7abb8&_ss=r*	*Polymer*
*Silc Pig Silicone Pigment*	*5g*	*NZ$60/100g*	*NZ$3*	*https://www.resincraft.co.nz/products/silc-pig-silicone-pigments?srsltid=AfmBOor1mQvFF87ywFuPy4DYixdWn9je4lWUnuOJ_619pnJqmJA1F237*	*Organic*
*M6 Bolts*	*3*	*NZ$0.5*	*NZ$1.5*	*https://www.blacksfasteners.co.nz/bst30406050*	*304 Stainless Steel*
*M6 Nuts*	*6*	*NZ$0.08*	*NZ$0.48*	*https://www.blacksfasteners.co.nz/n06z*	*304 Stainless Steel*

## 5 Build instructions

### 5.1 Mould Fabrication

The symmetric mould (*Symmetric Breast Phantom Mould*) was SLA 3D printed using a Phenom Noir printer using FormLab’s tough 2000 resin. The breast model (*Asymmetric Breast Model*). was 3D printed in 20% infill PLA and then sanded first with Flexovit P120 sandpaper followed by Flexovit P240 sandpaper to remove layer lines from printing. After these preparation steps, the model was thermoformed with 1 mm PETG plastic.

### 5.2 Plate Fabrication

The four plates (*Symmetric Breast Phantom Insert Plate, Symmetric Mould Top Plate*, *Asymmetric Breast Phantom Insert Plate* and *Asymmetric Mould Top Plate*) were laser cut on clear acrylic sheet with 4.5 mm thickness using a Thunderlaser MARS 120 laser cutter.

### 5.3 Phantom Fabrication

The proposed silicone breast phantom is constructed in several stages. The tumours are cured and shaped first. The skin layer is then constructed and cured before the adipose tissue is added. The step-by-step instructions for breast phantom fabrication are below, with integration of the stages. For development of a healthy (non-cancerous) phantom, neglect the steps involving tumour preparation and placement.

Extra equipment needed for the fabrication of silicone breast phantoms is listed below.

• Electronic scales
• Vacuum chamber
• Oven
• 5 mm leather punch
• Scalpel or sharp knife/blade
• Plastic cups
• Wooden sticks
• Thread

#### 5.3.1 Tumour Preparation

Tumours of 10 mm diameter were made in this example as this is the smallest size expected to be detected.

**Fig. 1:**
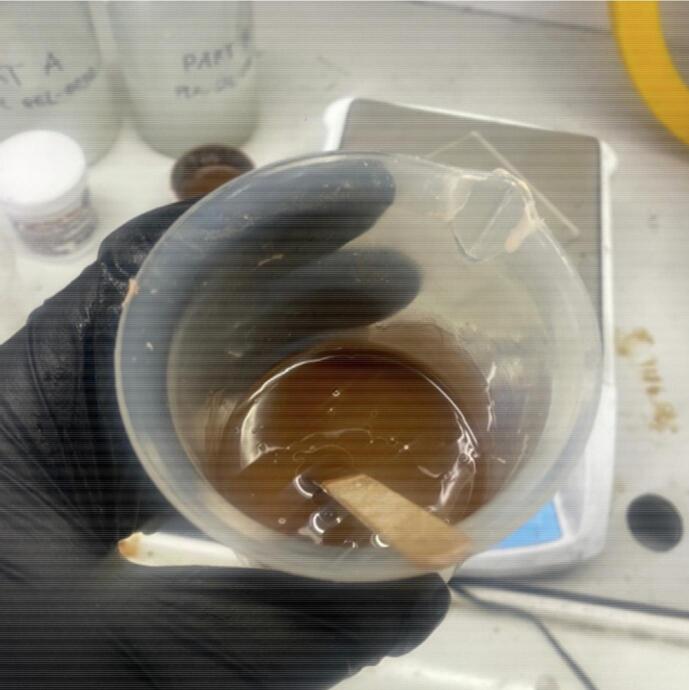
Tumour mixture.
• Place the cup in a vacuum chamber and pressurise to -28 InHg (Fig. 2). Leave in vacuum for 2 minutes until bubbles have subsided. Slowly release the pressure from the chamber and remove the cup.

• Spray a disposable plastic tray with Ease Release Mann 200, coating all internal surfaces.
• Combine 85 grams of Platsil Gel 0030 Silicone (1:1 ratio of Parts A and B) with 15 grams of PMX-200 Silicone in a plastic cup. Optionally, add 0.5 g of Silc Pig Silicone Pigment so the tumours can be seen through the adipose tissue. Stir well (Fig. 1).

**Fig. 2:**
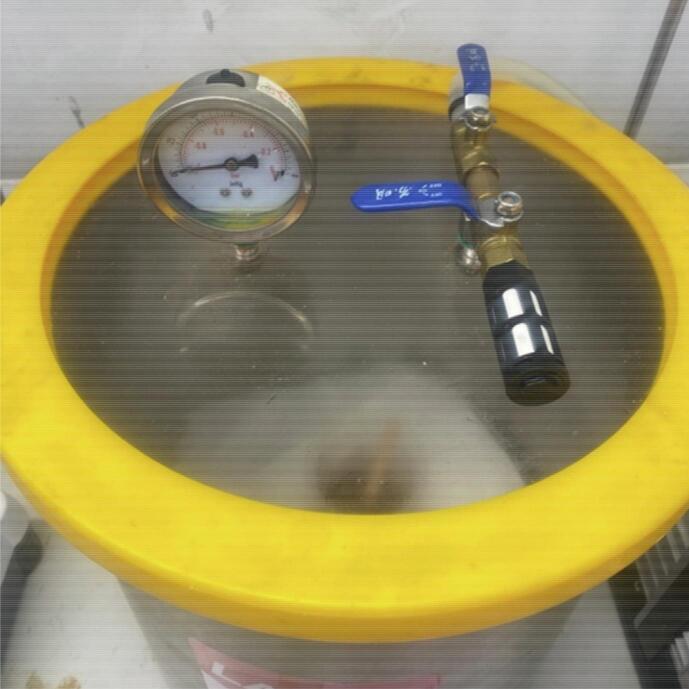
Use of vacuum chamber.
• Pour the solution into the disposable tray to a depth of 10 mm (Fig. 3).

**Fig. 3:**
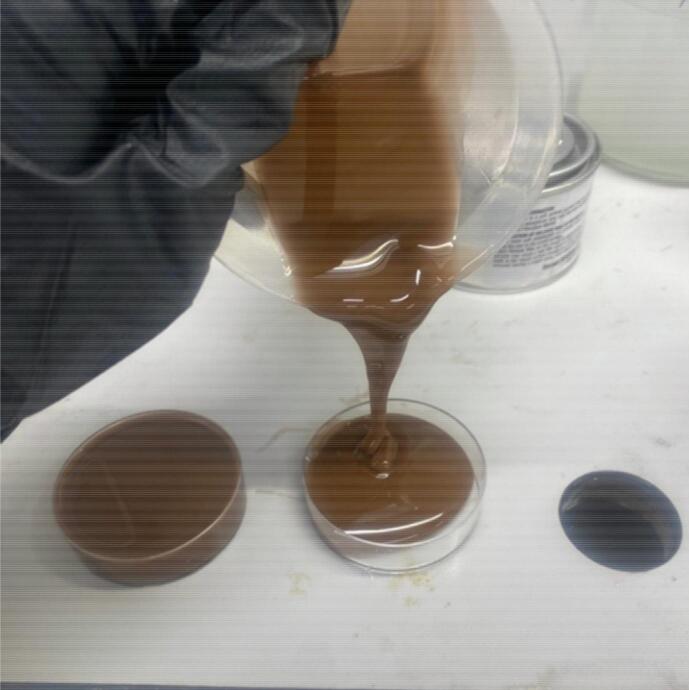
Trays of tumour material before curing.
• Cure in the oven at 60°C for 1 hour.
• Use a 5 mm leather punch to form a hole in the tumour material.
• Using a Scalpel, cut a rough 10 mm sphere around the hole (Fig. 4).

**Fig. 4:**
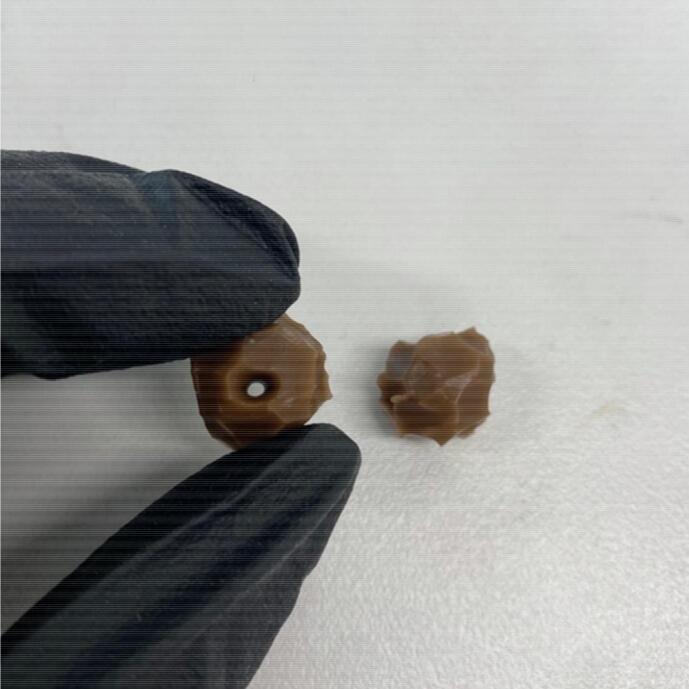
Completed tumours.

#### 5.3.2 Mould Preparation

• Apply a thin, even coat of Norski Mould Release Wax to the mould. Polish it into the surface with a cloth or paper towel.
• Place the mould in the oven at 60°C for five minutes to set the wax.
• Spray the mould with Ease Release Mann 200, being sure to coat all internal surfaces.

#### 5.3.3 Skin Layer Preparation

**Fig. 5:**
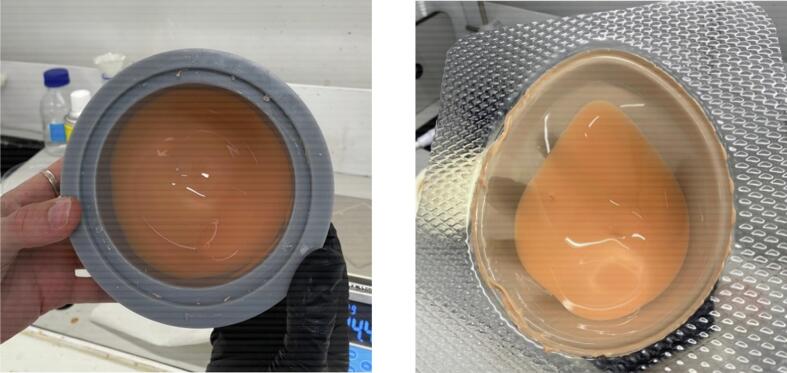
Skin layer from manual rotation (a) in symmetric mould and (b) in asymmetric mould.
• Cure the skin layer in the oven at 60°C for 15 minutes.
• With a scalpel or blade, cut off the excess skin material around the top of the mould (Fig. 6, Fig. 6).

• Combine 16 grams of Platsil Gel 0030 Silicone (1:1 ratio of Parts A and B) with 4 grams of PMX-200 Silicone in a plastic cup. Add 0.2 grams of Silc Pig Silicone Pigment in the desired shade. Stir well.
• Place the cup in a vacuum chamber and pressurise to -28 InHg. Leave in vacuum for 2 minutes until bubbles have subsided. Slowly release the pressure from the chamber and remove the cup.
• Pour the solution into the prepared mould. Rotate the mould manually to coat all surfaces (Fig. 5, Fig. 5). Place the mould upside down on a tray.

**Fig. 6:**
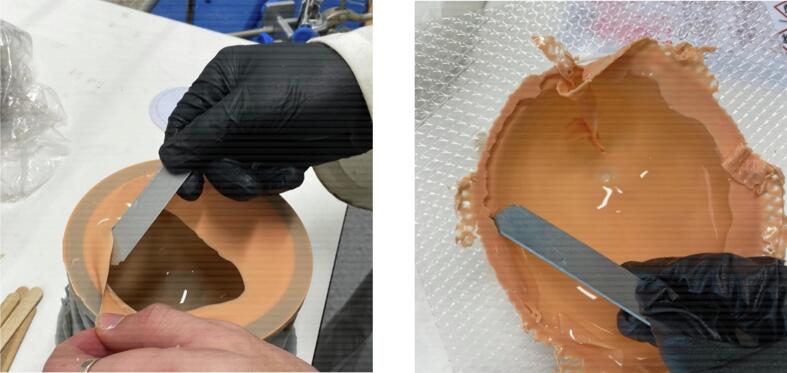
Removal of excess skin material (a) from symmetric mould and (b) from asymmetric mould.
• Repeat the previous steps four times to create another skin layer.
• On the last two repetitions, place the mould in the oven upside down on the tray for one minute. Remove and rotate to right side up to cure again in the oven for one minute. Rotate once more such that it is upside down, and cure in this position for the remaining 13 minutes.
• Cut off all the excess skin, including that on the top plate seat of the mould (Fig. 7, Fig. 7).

**Fig. 7:**
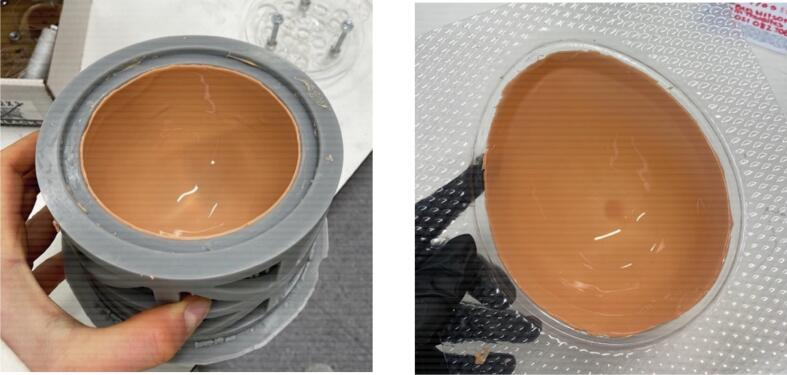
Completed skin layer (a) in symmetric mould and (b) in asymmetric mould.

#### 5.3.4 Adipose Tissue

**Fig. 8:**
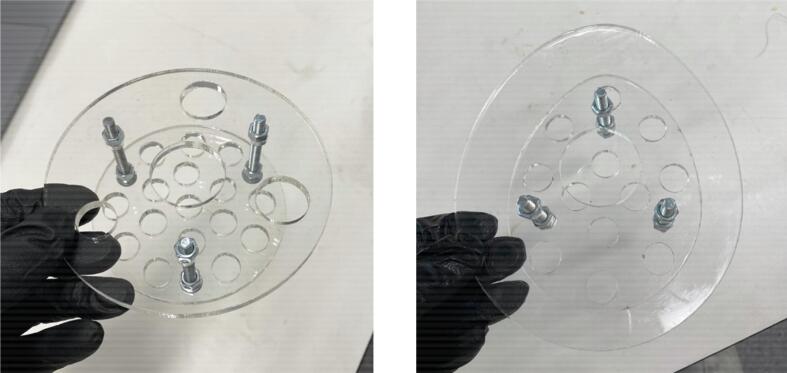
Insert plate and top plate assembly for (a) symmetric phantom and (b) asymmetric phantom.
• If including a tumour, suspend the tumour in the desired position with thread. Tie the thread to the top plate or bolts to secure its position (Fig. 9, Fig. 9).

• Combine 140 grams of Platsil Gel 0030 Silicone (1:1 ratio of Parts A and B) with 210 grams of PMX-200 Silicone in a plastic cup. Stir well.
• Place the cup in a vacuum chamber and pressurise to -28 InHg. Leave in vacuum for 2 minutes until bubbles have subsided. Slowly release the pressure from the chamber and remove the cup.
• Attach the insert plate to the top plate with three bolts and six nuts (Fig. 8, Fig. 8). Ensure that the nuts securing the insert plate position are tight. Place this in the mould over the cured skin layer, adjusting the top nuts to position the insert plate. The insert plate should sit 10 mm below the top surface of the mould.

**Fig. 9:**
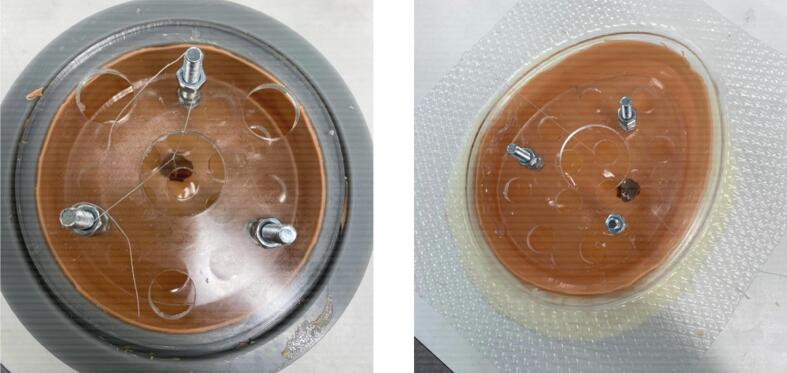
Plates and tumour positioned in (a) symmetric mould and (b) asymmetric mould.
• Pour the adipose tissue silicone mixture into the mould (Fig. 10, Fig. 10), being sure to pour until the top of the nuts on the insert plate are just covered. Adjust the tumour and insert plate positions if necessary.

**Fig. 10:**
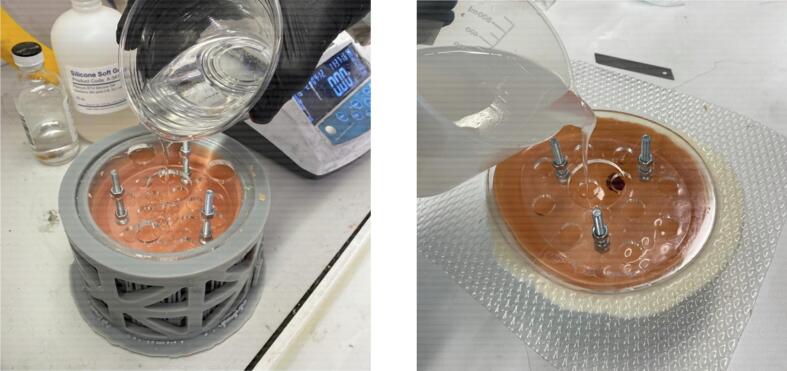
Addition of adipose material (a) in symmetric mould and (b) in asymmetric mould.
• Cure in the oven at 60°C for 2 hours.

#### 5.3.5 Removal

• Unscrew the top nuts and remove the top plate.
• Gently release the phantom from the mould.
• Cut off excess skin around the base with sharp scissors.

**Fig. 11:**
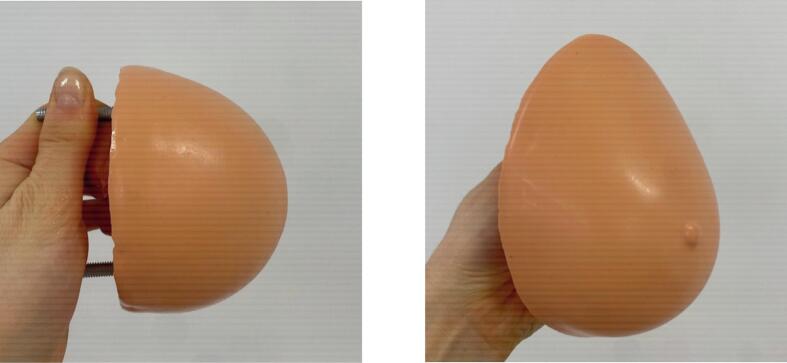
(a) Symmetric breast phantom and (b) asymmetric breast phantom.

The completed phantoms are pictured in Fig. 11a and Fig. 11b.

Safety concerns and risks associated with the fabrication of silicone phantoms are identified in Table 3.

**Table 3:** Risks involved with silicone breast phantom build.

**Risk**	**Information**	**Mitigation**
Fume inhalation	Mann Ease Release 200 contains hazardous ingredients.	• Use under a fume hood or in a well-ventilated area. • Refer to Material Safety Data Sheet when necessary.
Contamination of materials	Mann Ease Release 200, Platsil Gel-00 Silicone and PMX-200 Silicone fluid are susceptible to contamination.	• Wear appropriate PPE – safety glasses, long sleeves and gloves.
Hot surfaces	The oven temperature does not exceed 60 degrees Celsius for the purpose of curing the silicon in the breast phantoms. It is unlikely to cause harm to the operator.	• Exercise care when placing and removing items from the oven. • Use appropriate PPE.

## 6 Operation instructions

**Fig. 12:**
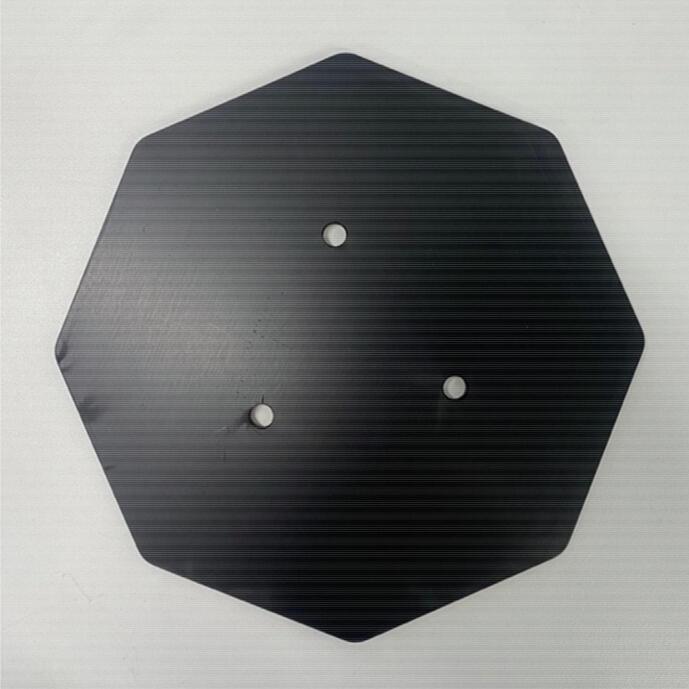
Silicone Breast Phantom Base Plate.

Silicone breast phantoms are stored on plates for protection and to minimise wear and tear. The plates (Fig. 12) also allow for easy suspension of the phantoms on the DIET machine. The plate design is attached as a design file (*Base Plate*). The plates were cut from 5 mm aluminium sheet and anodised with a 20-micron matte black finish. Breast phantoms are secured to the plates with nuts.

**Fig. 13:**
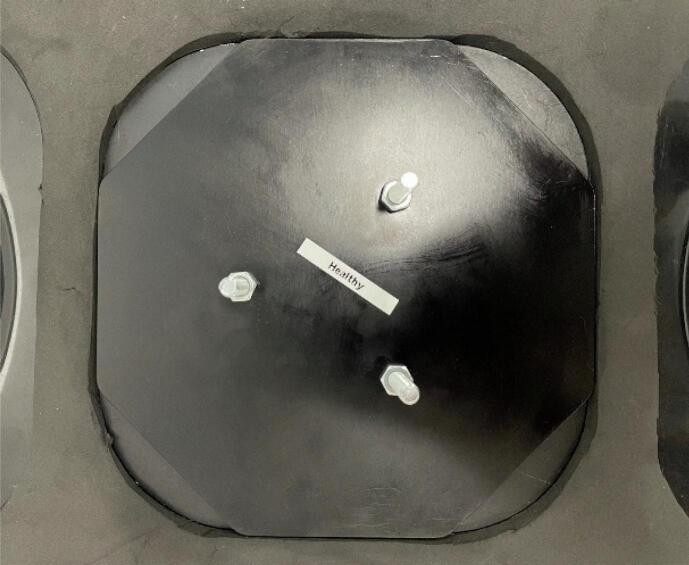
Placement of phantom in DIET machine.

**Fig. 14:**
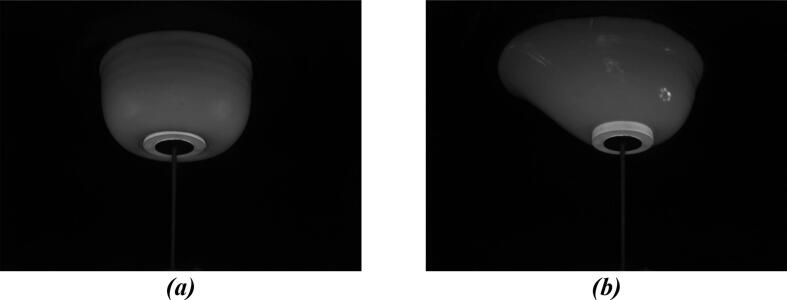
(a) Camera view of symmetric breast phantom and (b) camera view of asymmetric breast phantom.

The phantom on the base plate can easily be suspended in the DIET machine as pictured (Fig. 13**,** Fig. 14, Fig. 14).

## 7 Validation and characterization

To validate the mechanical similarities of the Silicone Breast Phantoms to real breasts, compression testing was performed with an MTS test machine. Testing was performed in alignment with ASTM D575-91 and ASTM D1056-20 standards with sample disks of 30 mm diameter and 12.5 mm height. Specific test parameters are detailed in Table 4.

**Table 4:** Compression testing parameters.

Load Cell	100 Newtons
Pre-load	0.1 Newtons
Speed	0.5 mm/minute
Stop Condition	25% Strain

**Fig. 15:**
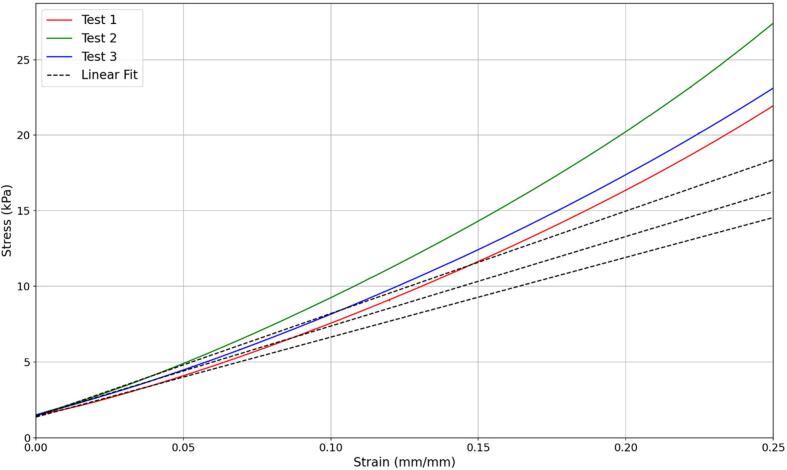
Stress strain curve with linear regression for a compression test using a sample with 85% silicone soft gel and 15% silicone oil.

The compression testing gave force and displacement data for each sample tested. These curves were transformed to stress and strain. The elastic modulus was determined by finding the gradient of the linear region between zero and five percent strain. The stress strain curves for one of the tests can be seen in Fig. 15 and the average elastic moduli for all samples can be seen in Table 5. In the DIET machine a strain of 1-2% is applied and so this is the linear region that the elastic modulus is being found from. The stress strain curve showing this detain can be seen in Fig. 16a**.**

**Table 5:** Elastic moduli of different silicone ratios.

Composition (Soft gel:Silicone oil)	Elastic Modulus (kPa)
100:0	117.4
90:10	91.8
85:15	59.9
80:20	57.8
45:55	18.7
40:60	15.3
35:65	10.5

**Fig. 16a:**
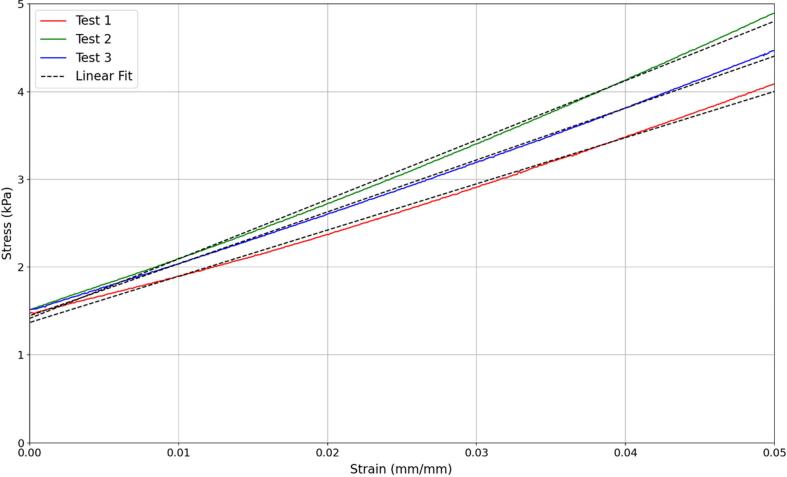
Stress strain curve from Fig. 15 as a close-up on the 5% strain region that is relevant to the use case.

Several studies have been conducted to determine the elastic properties of healthy and cancerous breast tissue. The results of these largely show that specific values are variable with consistent results not existing. However, all studies show a similar value for the ratio of cancerous and healthy fat tissue. A review of different sources values for different types of breast tissue can be seen in Table 6 [24].

**Table 6:** A summary of the results from mechanical testing of ex vivo breast tissue [24].

*Pre-strain*	*Normal fat tissue (kPa)*	*Normal Glandular tissue (kPa)*	*DCIS Tumour(kPa)*	*IDC Tumour(kPa)*	*Reference*
*5%*	*18-22*	*28-35*	*22-26*	*106-112*	[25]
*20%*	*20-24*	*48-66*	*291-307*	*558-460*	[25]
*1%*	*4.8*	*17.5*	*71.2*	*47.1*	[26]
*15%*	*17.4*	*271.8*	*2162*	*1366.5*	[26]
*5%*	*3.25*	*3.24*	*16.38*	*10.4-42.5*	[27,28]
*NA*	*5*	*50*	*100-5000*	*100-5000*	[29]
*NA*	*1*	*1*	*3.5*	*10*	[30]
*0-0.2 Stress*	*0.7*	*0.8*	*3.4*	*11.5*	[31]
*1.0-1.2 Stress*	*17.3*	*15.4*	*15.6*	*27*	[31]
*0-0.2 Stress*	*0.69*	*0.73*	*5.25*	*13.82*	[32]
*1.0-1.2 Stress*	*19.08*	*16.99*	*16.15*	*30.5*	[32]

There is a large amount of variability in the quoted elastic properties. Healthy fat tissue ranges from 0.69 to 20 kPa and tumour tissue ranges from 3.4 to 2162 kPa. For each study the tumour tissue was 4-10 times stiffer than the normal fat tissue. The significant variability makes it difficult to determine the exact properties of breast tissue for a phantom. This variability is also likely due, at least in part, to the mechanical testing scenarios used such as different types of preload compression, different experimental conditions, tissue heterogeneity, and other measurement errors [33]. In addition, the mechanical properties of breast tissues differ between individuals and over time due to the variability in anatomy, hormone levels, age and physiological condition. In particular, the impact between 5% and 20% pre-compression can be seen in Table 7 [25,34]. As a result, the goal was to match the ratio of cancerous tissue to healthy fat tissue, while being within the range of values quoted by the literature.

**Table 7:** A comparison of elastic moduli with different amounts of precompression at a loading frequency of 1 Hz [25].

*Breast Tissue Type*	*Tissue Elastic Modulus (kPa)*	
	*5% precompression*	*20% precompression*
*Normal Fat*	*19*±*7*	*20*±*6*
*Normal Glandular Tissue*	*33*±*11*	*57*±*19*
*Fibrous Tissue*	*107*±*31*	*232*±*60*
*Ductal Carcinoma*	*25*±4*8*	*301*±*58*
*Invasive ductal Carcinoma*	*93*±*33*	*490*±*112*

The healthy fat tissue selected in this research had an elastic modulus of 15.3 kPa and the tumour tissue had an elastic modulus of 59.9 kPa. These values fit the ranges and give a tumour to healthy tissue ratio of 4 times, which remains conservative and realistic. The process presented is also generalisable to other specific stiffness values for each major component and any reasonable ratio desired.

Smooth, spherical tumours exhibited poor connectivity to the adipose tissue [19,35]. To better integrate tumours into the breast phantoms, a donut shape was employed. This shape allows some adipose tissue to flow through the centre of the tumour providing greater connectivity and load or motion transfer between the tumour inclusion and surrounding tissue mimicking phantom material. The outer surface of the donut-shaped tumours is rough, rather than smooth, which also increases connectivity to the surrounding adipose tissue in this silicone phantom.

Tumors of 10 mm were used as it is near the smallest size detected by the DIET device to date and half of the approximately ∼20 mm average tumour size detected in mammography. These tumour inclusions were placed at varying depths in different silicone breast phantoms. In symmetric phantoms, the tumours were placed on the same side but at varying depths as shown by the top-view in Fig. 16b. In asymmetric phantoms, the tumours were placed at the same depth but with varying position as shown by the top view of the phantom in Fig. 17.

**Fig. 16b:**
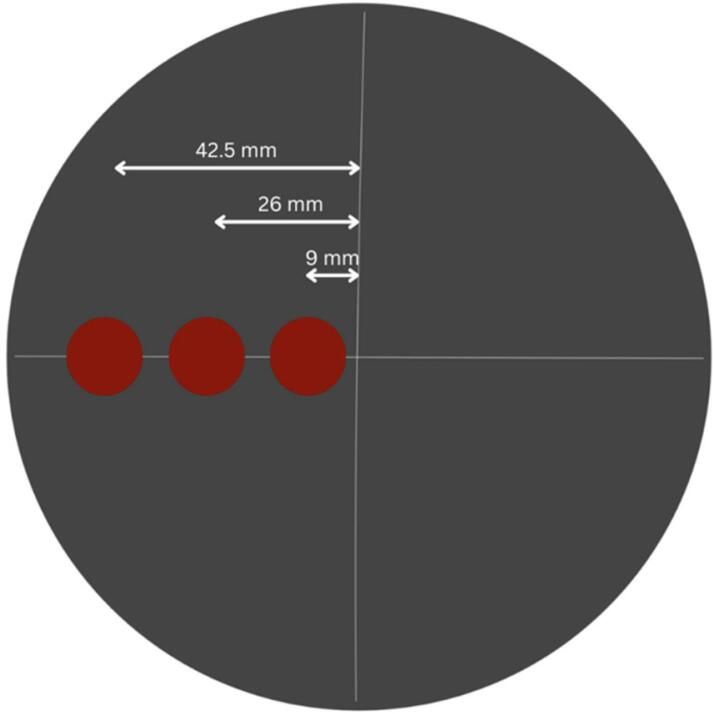
Tumour placement in symmetric phantoms.

**Fig. 17:**
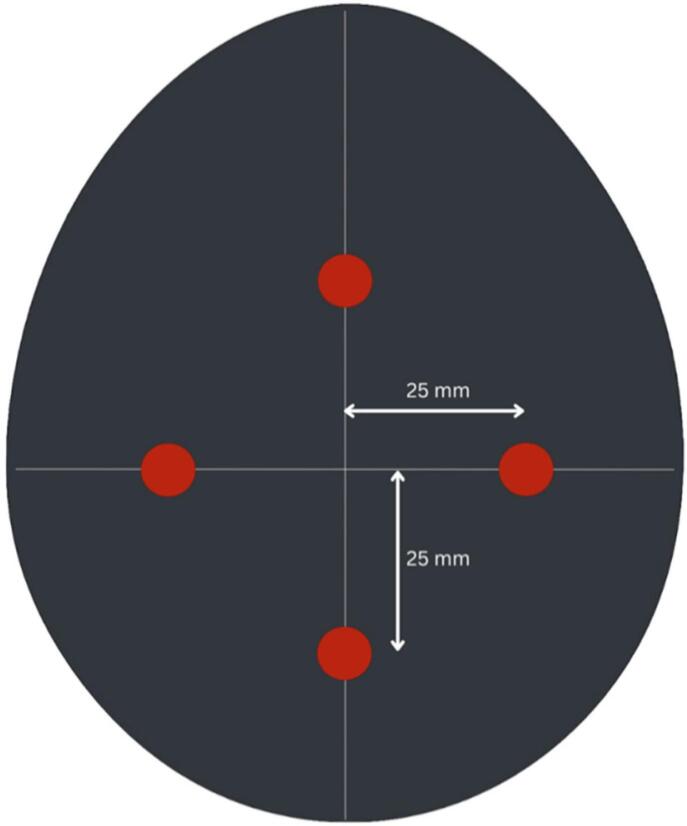
Tumour placement in asymmetric phantoms.

## Ethics statements

This research did not involve any work that required ethics approval or consent.

## CRediT authorship contribution statement

**Francesca Gallaway:** Writing – original draft, Validation, Methodology, Conceptualization. **Henry W. Hall:** . **Matthew Walker:** Validation, Methodology, Conceptualization. **Chloe Tattersfield:** Conceptualization. **Stephanie Post:** Conceptualization. **Jessica Fitzjohn:** Writing – review & editing, Supervision. **Cong Zhou:** Writing – review & editing, Supervision. **J.Geoffery Chase:** .

## Declaration of competing interest

The authors declare that they have no known competing financial interests or personal relationships that could have appeared to influence the work reported in this paper.
